# *Lactobacillus zeae* Protects *Caenorhabditis elegans* from Enterotoxigenic *Escherichia coli*-Caused Death by Inhibiting Enterotoxin Gene Expression of the Pathogen

**DOI:** 10.1371/journal.pone.0089004

**Published:** 2014-02-18

**Authors:** Mengzhou Zhou, Hai Yu, Xianhua Yin, Parviz M. Sabour, Wei Chen, Joshua Gong

**Affiliations:** 1 State Key Laboratory of Food Science and Technology, Jiangnan University, Wuxi, Jiangsu, China; 2 Guelph Food Research Centre, Agriculture and Agri-Food Canada, Guelph, Ontario, Canada; Massachusetts General Hospital, Harvard Medical School, United States of America

## Abstract

**Background:**

The nematode *Caenorhabditis elegans* has become increasingly used for screening antimicrobials and probiotics for pathogen control. It also provides a useful tool for studying microbe-host interactions. This study has established a *C. elegans* life-span assay to preselect probiotic bacteria for controlling K88^+^ enterotoxigenic *Escherichia coli* (ETEC), a pathogen causing pig diarrhea, and has determined a potential mechanism underlying the protection provided by *Lactobacillus*.

**Methodology/Principal Findings:**

Life-span of *C. elegans* was used to measure the response of worms to ETEC infection and protection provided by lactic acid-producing bacteria (LAB). Among 13 LAB isolates that varied in their ability to protect *C. elegans* from death induced by ETEC strain JG280, *Lactobacillus zeae* LB1 offered the highest level of protection (86%). The treatment with *Lactobacillus* did not reduce ETEC JG280 colonization in the nematode intestine. Feeding *E. coli* strain JFF4 (K88^+^ but lacking enterotoxin genes of *estA*, *estB*, and *elt*) did not cause death of worms. There was a significant increase in gene expression of *estA*, *estB*, and *elt* during ETEC JG280 infection, which was remarkably inhibited by isolate LB1. The clone with either *estA* or *estB* expressed in *E. coli* DH5α was as effective as ETEC JG280 in killing the nematode. However, the *elt* clone killed only approximately 40% of worms. The killing by the clones could also be prevented by isolate LB1. The same isolate only partially inhibited the gene expression of enterotoxins in both ETEC JG280 and *E. coli* DH5α *in-vitro*.

**Conclusions/Significance:**

The established life-span assay can be used for studies of probiotics to control ETEC (for effective selection and mechanistic studies). Heat-stable enterotoxins appeared to be the main factors responsible for the death of *C. elegans*. Inhibition of ETEC enterotoxin production, rather than interference of its intestinal colonization, appears to be the mechanism of protection offered by *Lactobacillus*.

## Introduction

Probiotics are live microorganisms that can confer a healthy benefit on their host [1]. They have long been used in food animal production and widely considered to be a potential alternative to dietary antibiotics. Lactobacilli are often used as probiotic bacteria. Their beneficial effects include reduction in colonization of animal intestines by pathogenic bacteria, improvement of animal performance, and enhancement of the animal immune system [2], [39], [40], [41]. Selection of *Lactobacillus* isolates with probiotic properties from a large number of bacteria is critical in developing effective probiotics and is largely limited by the high cost and low efficiency associated with the use of target food-producing animals for the selection [3]. *Caenorhabditis elegans* is a small free-living soil nematode that has been extensively used as an experimental *in-vivo* system for biological studies because of its small size, short generation time, suitability for genetic analysis. In particular, *C. elegans* has been used to study pathogen and host interactions of various bacterial pathogens, such as *Pseudomonas aeruginosa* [4], *Salmonella enterica* [5]–[7], *Staphylococcus aureus* [8], and *Enterococcus faecalis* [9]. In addition, the nematode has become increasingly used for screening antimicrobials, including preselecting probiotic bacteria for *Salmonella* control [7], [11], [12]. Moreover, it provides a useful tool for mechanistic studies of probiotic effects. Yet, no studies on using *C. elegans* to preselect probiotic candidates for enterotoxigenic *Escherichia coli* (ETEC) control have been reported thus far, although ETEC is one of the most important etiological agents causing diarrhea in piglets, leading to significant losses in swine production [13]. In addition, more evidence is still required to elucidate the molecular mechanisms underlying probiotic effects for better understanding and utilization of probiotics.

Diarrhea, a major cause of mortality to newly weaned pigs, is often caused by ETEC and jeopardizes swine production [14]. Porcine ETEC strains are characterized by the production of specific adhesins and enterotoxins. The fimbrial adhesins K88 (F4) and heat-stable (ST) and heat-labile (LT) enterotoxins have been identified as important factors contributing to diarrheal diseases [15], [16]. The swine industry has relied largely on prophylactic use of antibiotics to control ETEC and its associated diarrhea. However, there is a growing concern over the practice due to the wide spread of antibiotic-resistance in zoonotic bacterial pathogens, which poses a threat to public health. Thus, strategies to control the pathogen by a natural alternative to antibiotics are urgently required to reduce the prophylactic use of antibiotics in swine production. In addition, elucidation of the mechanisms underlying the function of these alternatives to antibiotics remains critical in supporting the development and application of the alternatives. The objective of the present study was to establish a *C. elegans* life-span assay model to measure the response of worms to ETEC infection, which allows a quick assessment of the protection offered by *Lactobacillus* and studies of microbe-host interactions. In addition, the mechanism of protection provided by *Lactobacillus* has been explored.

## Results

### Establishment of a Life-span Assay of *C. elegans* Infected with ETEC

**Figure 1:**
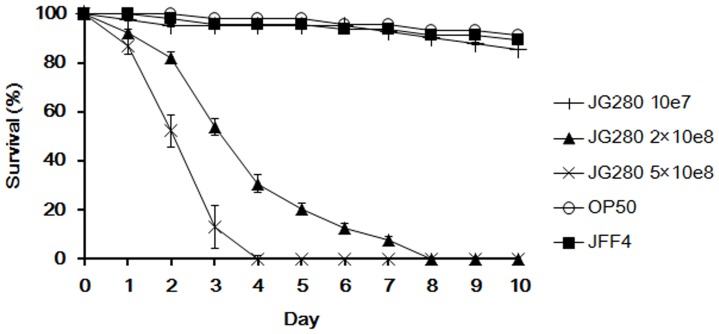
Establishment of a life-span assay of *C. elegans* infected with K88^+^ ETEC strain JG280. The life-span is expressed as survival of *C. elegans* during the assay after infection with JG280 at different cell concentrations. In the assay, worms were fed one of the following for 10 days: ○, *E. coli* OP50 (food for *C. elegans*) at 10^8^ CFU/ml; +, JG280 at 10^7^ CFU/ml; ▪, JFF4 at 5×10^8^ CFU/ml; ▴, JG280 at 2×10^8 ^CFU/ml; ×, JG280 at 5×10^8 ^CFU/ml.

To establish a *C. elegans* life-span assay capable of measuring the response of worms to ETEC infection, K88^+^ ETEC strain JG280 at different concentrations, ranging from 10^7^ to 10^9^ colony forming units (CFU) per ml, was used in the assay instead of *E. coli* OP50 that is normally used as food to maintain the nematode when *C. elegans* reaches the L4 stage. The effect of JG280 on the death of *C. elegans* appeared to be dose-dependent. At 10^7 ^CFU/ml, JG280 was similar to OP50 and showed no significant effect on the viability of worms after ten days of incubation (Fig. 1). However, the life span of worms was dramatically decreased when the concentration of JG280 was increased to 2×10^8 ^CFU/ml, in which almost all worms were dead after eight days of incubation. At 5×10^8 ^CFU/ml of JG280, the life span of *C. elegans* was further shortened to four days. The effect of K88^+^ strain JFF4 (containing no enterotoxin genes) on the death of *C. elegans* was also examined. Similar to OP50, JFF4 caused no significant death of *C. elegans* at concentrations up to 5×10^8 ^CFU/ml. Hence, the assay with *C. elegans* infected by JG280 was used to assess selected lactic acid-producing bacterial (LAB) isolates for their protection against the death of worms caused by JG280 infection.

### Effect of LAB Isolates on the Protection of *C. elegans* against ETEC JG280 Infection

Thirteen LAB isolates selected previously from pigs and chickens by our group were assessed for their ability to protect *C. elegans* from JG280-caused death. The isolates varied in their ability to protect live worms with survival rates from 22 to 79% on the last day (day 10) of the assays (Table 1). While isolates CL11 and S20 demonstrated the lowest protection (22 and 23% survival of worms, respectively), isolates S33, S64, and LB1 provided the highest protection with approximately 70, 77, and 79% survival of the worms, respectively, at the end of assays. The negative control (fed *E. coli* OP50 only) and ETEC reference groups (treated with *E. coli* OP50 followed by ETEC strain JG280) normally had approximately 91% and near 0% survival of worms, respectively.

**Table 1:** Statistical analysis of the protection effect of lactic acid-producing bacterial isolates on *C. elegans* infected with ETEC JG280^a^.

						*P*-value^f^	
Treatment^b^	Origin^c^	Putative identity^d^	Survival (%)	95% CI (± %)	DT50^e^ (day)	L+J vs *E. coli* OP50	L+J vs E+J
E+JG280			0.00	4.67	3.70	<0.0001	
*E. coli* OP50			91.11	5.67	>10		<0.0001
CL10	P	*Lactobacillus acidophilus*	39.47	8.13	6.03	<0.0001	<0.0001
CL11	P	*Lactobacillus casei*	23.26	6.34	3.78	<0.0001	0.046
CL12	P	*Lactobacillus amylovorus*	39.02	7.91	7.52	<0.0001	<0.0001
K16	P	*Pediococcus pentosaceus*	67.74	3.21	>10	0.004	<0.0001
K30	P	*Lactobacillus sp.*	35.71	8.26	6.01	<0.0001	<0.0001
K67	P	*Lactobacillus reuteri*	30.77	3.12	4.99	<0.0001	0.006
S8	P	*Lactobacillus plantarum*	34.88	5.41	5.34	<0.0001	0.001
S20	P	*Lactobacillus johnsonii*	21.82	3.63	5.28	<0.0001	0.001
S33	P	*Pediococcus pentosaceus*	70.00	5.94	>10	0.005	<0.0001
S64	C	*Lactobacillus reuteri*	77.42	5.75	>10	0.049	<0.0001
S65	P	*Lactobacillus plantarum*	61.29	11.17	>10	<0.001	<0.0001
S66	P	*Lactobacillus plantarum*	56.82	10.12	>10	<0.0001	<0.0001
LB1	C	*Lactobacillus zeae*	78.57	7.28	>10	0.093	<0.0001
Heat-killed LB1			22.12	5.67	>10	<0.0001	0.047

a Summary of two or more separate experiments. Survival of worms on the last day (day 10) of the assays with 95% confidence interval (CI) was estimated with the Kaplan-Meier survival analysis.

b E+JG280: treatment with *E. coli* OP50 and then with ETEC K88 strain JG280. In the assays with LAB isolates, the nematode was firstly treated with a LAB isolate and then with ETEC JG280.

c C, isolates from chickens; P, isolates from pigs.

d Putative species identity was determined by BLAST analysis of sequences of 16S rRNA genes. Sequence similarities between the isolates and the 16S rDNA database sequences were 98 to 100%. Among the thirteen isolates, CL10, CL11, CL12, S64 and LB1 have been reported previously (7).

e DT50, the time at which half of the worms were dead.

f Comparisons of survival curves. L+J, *C. elegans* was treated with *Lactobacillus* and then JG280. L+J vs *E. coli* OP50, the statistical difference between the group of *C. elegans* treated with *Lactobacillus* followed by JG280 and the group of *C. elegans* treated with *E. coli* OP50 only (control group). L+J vs E+J, the statistical difference between the group of *C. elegans* treated with *Lactobacillus* followed by JG280 and the group of *C. elegans* treated with *E. coli* OP50 followed by JG280.

### Effect of *Lactobacillus* on the Colonization of ETEC Strain JG280 in the Intestine of *C. elegans*

**Figure 2:**
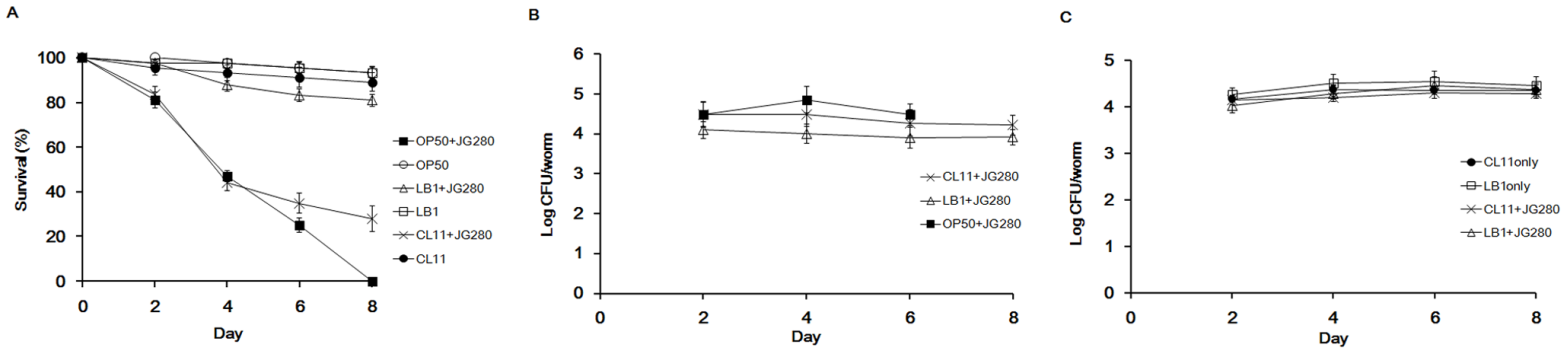
Effect of feeding isolates LB1 (*Lactobacillus zeae*) and CL11 (*Lactobacillus casei*) on the survival of *C. elegans* infected with ETEC JG280 and on bacterial colonization of the nematode intestine. (A) Survival (%) of *C. elegans* in the presence or absence of *Lactobacillus*. (B) Colonization of ETEC JG280 in the intestine of worms. (C) Colonization of *Lactobacillus* in the intestine of worms. Control worms were fed *E. coli* OP50 only, either isolate LB1 or CL11 at 10^8 ^CFU/ml for 8 days. In other treatments, worms were first fed either *E. coli* OP50 or *Lactobacillus* (isolate LB1 or CL11) at 10^8^ CFU/ml for 18 h and then ETEC JG280 for the remaining days. Treatments:○, *E. coli* OP50 only; ▪, *E. coli* OP50 and then ETEC JG280; □, isolate LB1 only; •, isolate CL11 only; △, isolate LB1 and then JG280; × isolate CL11 and then JG280. The curves of two treatments (□ and ○) were almost overlapped in Panel A.

**Figure 3:**
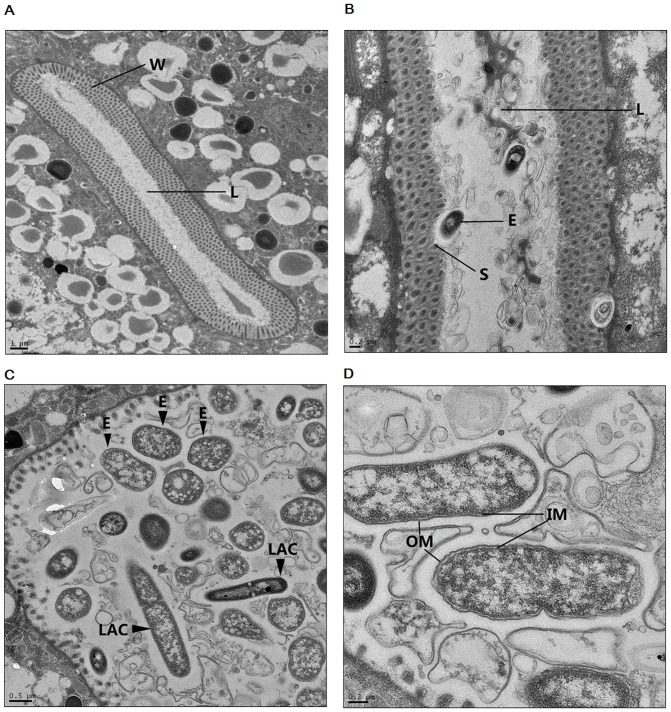
TEM images showing the intestine of *C. elegans* and the colonization of ETEC and *L. zeae* LB1 in the nematode intestine. (A) Cross section of the whole intestine of *C. elegans*. L: the lumen of intestine; W: the wall of intestine. (B) Colonization of ETEC JG280 in the intestine with a bacterial cell attached to the intestinal surface. E: ETEC JG280 cell; S: inner surface of the intestine; L: the lumen of intestine. (C) Co-existence of ETEC JG280 and *L. zeae* LB1 in the intestinal lumen of worms. E: ETEC JG280 cells; LAC: *L. zeae* LB1 cells. (D) Image of ETEC JG280 cells showing the inner and outer members of G-negative bacterium. IM: inner member; OM: outer member. The size of images is indicated by the scale bars.

The colonization of JG280 and two *Lactobacillus* isolates (*L. zeae* LB1 and *L. casei* CL11) in the intestine of *C. elegans* during the life-span assays was examined. While isolate LB1 was able to effectively protect the nematode, the protection by isolate CL11 was far less efficient (Fig. 2A). The colonization level of JG280 in the nematode intestine remained at approximately log_10_ 4–5 CFU/worm during the assay regardless of the absence or presence of isolates LB1 or CL11 (Fig. 2B). Similarly, both *Lactobacillus* isolates had a similar level of colonization (log_10_ 4–5 CFU/worm) in the nematode intestine during the assay regardless of the absence or presence of JG280 (Fig. 2C). The co-colonization of ETEC and *Lactobacillus* in the nematode intestine was also verified by transmission electron microscopy analysis, and both JG280 and LB1 cells were visible in the intestine (Fig. 3). These data indicate that intestinal colonization of JG280 may not be the major factor causing the death of *C. elegans* and the *Lactobacillus* isolates had no effect on the colonization of the nematode intestine by JG280.

### Effect of *Lactobacillus* on the Enterotoxin Gene Expression of ETEC JG280 Recovered from Infected *C. elegans*

**Figure 4:**
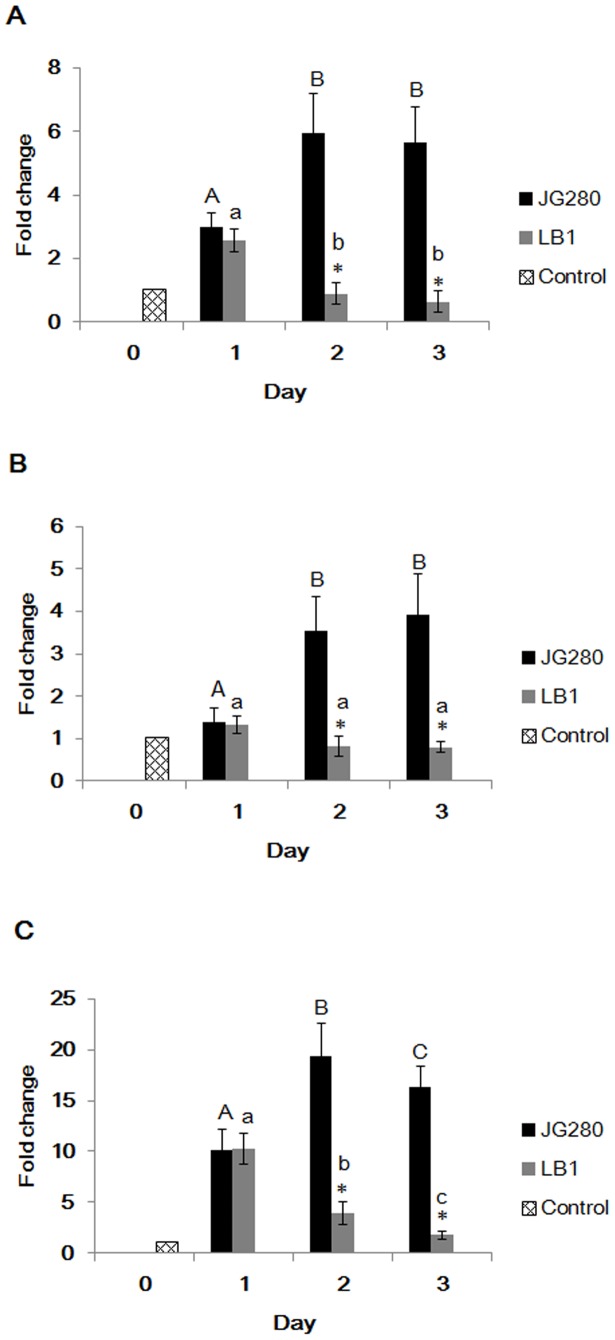
Gene expression of enterotoxins in ETEC strain JG280 during the life-span assay in the presence or absence of *L. zeae* LB1. The baseline (Gridline bar) is the level of gene expression of three enterotoxins in ETEC JG280 just before mixing with *C. elegans* (day 0). (A), (B), and (C) represent the expression level of *estA* (STa), *estB* (STb), and *elt* (LT), respectively, produced by ETEC JG280, in the absence (black bars) or presence of *L. zeae* LB1 (grey bars) on day 1, 2, and 3. Relative expression was determined using the 2^−ΔΔCt^ method as the ratio of gene transcript level of each time point to zero time point ETEC JG280 (day 0 before inoculation of *C. elegans*) and expressed as fold changes. Data are presented as mean ± S.D. Means marked with different letters (a, b, c,) are significantly different at *P* values of <0.05 within the ETEC JG280 group. Means marked with different letters (A, B, C) are significantly different at *P* values of <0.05 within the *L. zeae* LB1 group. *Represents significant difference from the ETEC JG280 group within the same day.

To determine the mechanism of *Lactobacillus* in protecting *C. elegans* against ETEC infection, the *invivo* gene expression of the three enterotoxins (*estA*, *estB*, and *elt*) of JG280 in first three days of life-span assays was examined in the presence and absence of *Lactobacillus*. When *C.elegans* was infected with the ETEC strain only, the expression of all three enterotoxin genes of JG280 was significantly enhanced, particularly on days 2 and 3, compared to their expression at the beginning of the assay (day 0) (Fig. 4). The enhanced enterotoxin gene expression coincided with a significant increase in the death of *C.elegans* on days 2 and 3 (Fig. 1). However, the enhanced gene expression of the three enterotoxin genes in these two days was remarkably reduced by the treatment with isolate LB1 (Fig. 4) that also prevented the nematode from JG280-induced worm death (Table 1). These results suggest that entertoxins are involved in the death of *C. elegans* caused by the ETEC infection.

**Figure 5:**
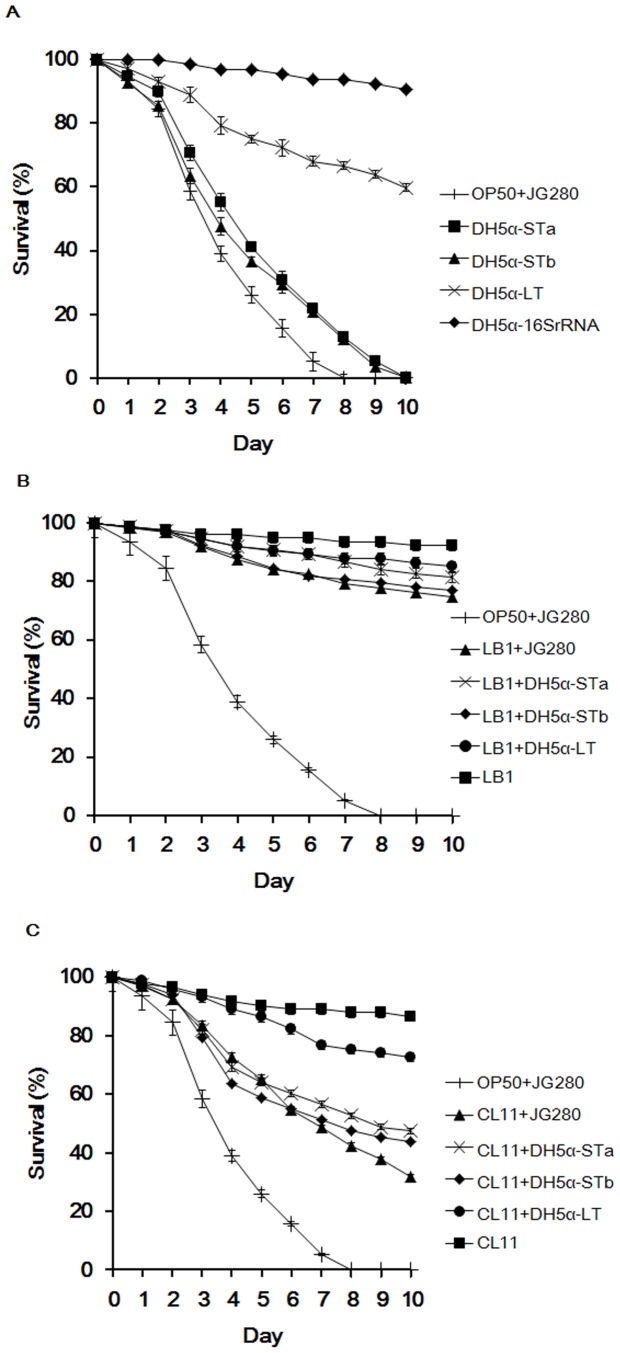
Effect of individual clones harboring an enterotoxin gene from ETEC JG280 on the life span of *C. elegans* in the absence or presence of *Lactobacillus*. (A) Effect of individual enterotoxin clones on the life span of *C. elegans.* Worms were fed one of the following for 10 days: ♦,clone DH5α-16SRNA at 2×10^8 ^CFU/ml; ×, clone DH5α-LT at 2×10^8 ^CFU/ml; ▪, clone DH5α-STa at 2×10^8 ^CFU/ml; ▴, clone DH5α-STb at 2×10^8 ^CFU/ml; +, OP50 then ETEC JG280 at 2×10^8 ^CFU/ml. (B) and (C) Effect of isolates LB1 (*L. zeae*) and CL11 (*L. casei*)) on the life span of *C. elegans* infected with individual enterotoxin clones. Worms treated with ETEC JG280 isolate LB1, or CL11 only served as the controls for corresponding treatments. The concentration of all bacterial cultures used for the assays was 2×10^8 ^CFU/ml. In the treatment groups, worms were treated initially with a *Lactobacillus* isolate at 10^8^ CFU/ml for 18 h and then with an individual clone or ETEC JG280 (2×10^8 ^CFU/ml) for the remaining days. All the assays were kept for 10 days. ▪, isolate LB1 or CL11 only; •, isolate LB1 or CL11 and then clone DH5α-LT; ×, isolate LB1 or CL11 and then clone DH5α-STa; ♦, isolate LB1 or CL11 and then clone DH5α-STb; ▴, isolate LB1 or CL11 and then ETEC JG280; +, OP50 and then ETEC JG280.

To further determine the role of the three ETEC enterotoxins in causing worm death and the protective mechanism provided by *Lactobacillus*, each enterotoxin expression in *E. coli* DH5α (a non-pathogenic *E. coli* isolate harboring no enterotoxin genes) was tested for its effect on the life span of *C. elegans* in the absence and presence of *Lactobacillus*. As demonstrated in Fig. 5A, both heat-stable enterotoxin clones (DH5α-STa and DH5α-STb) and ETEC strain JG280 had a similar effect on the death of the nematode and all worms had died at the end of the assay (day 10). However, the heat-labile enterotoxin clone (DH5α-LT) killed only 40% worms by the end of assay. Little death of *C. elegans* was caused by the clones harboring a 16SrRNA gene (DH5α-16SrRNA, Table S1) or one of the heat-stable enterotoxin genes (DH5α-opSTa, or DH5α-opSTb, Table S1) with an opposite orientation in the vector and unable to be expressed (data not shown).

Similar to the protection offered by isolate LB1 or CL11 to ETEC JG280-caused death of *C. elegans*, each of the isolates provided a comparable degree of protection to the nematode from death induced by each of the enterotoxin clones (DH5α-STa, DH5α-STb, and DH5α-LT) (Fig. 5B and 5C). Isolate LB1 was able to improve the survival rate of worms up to 80% regardless of the infection caused by strain JG280 or by any of the enterotoxin clones (Fig. 5B). In the same experiments, the treatment with isolate CL11 increased the nematode survival rate to approximately 40–50% from near 0% regardless of the strains used for treatment strain, either JG280 or one of the heat-stable exterotoxin clones (DH5α-STa and DH5α-STb) (Fig. 5C). When the nematode was infected with the heat-liable enterotoxin clone (DH5α-LT) after the treatment with isolate CL11, approximately 70% of the worms were survived to the end of the assay, at least 10% improvement in the survival rate compared to the assay without the CL11 treatment.

**Figure 6:**
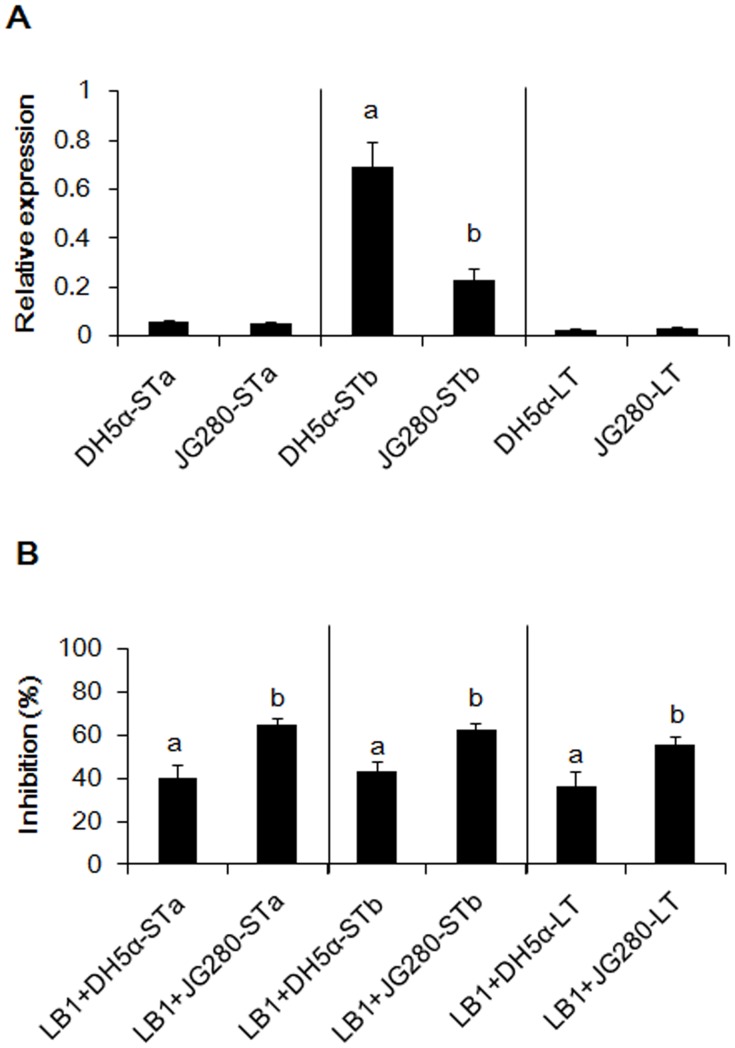
Comparison of the enterotoxin clones with ETEC JG280 with regard to *in-vitro* expression of enterotoxin genes in the absence or presence of *L. zeae* LB1. (A) Relative expression of *estA*, *estB*, and *elt* genes in the absence of isolate LB1. The data of relative expression were log_10_ 2 transformed to acquire a normal distribution after normalization with the housekeeping gene, *gapA*, as a reference. Data are presented as relative expression changes calculated by comparing three enterotxin expression levels with the housekeeping gene. A value of >1 represents up-regulation; a value of <1 represents down-regulation. (B) Inhibition of gene expression (relative expression) of *estA*, *estB*, and *elt* by isolate LB1. The relative expression of each toxin gene in the absence of LB1 represented 0% inhibition. The bars denoted with different letters represent significant differences (*P*<0.05).

*In-vitro* expression of enterotoxin genes (*estA*, *estB*, and *elt*) in ETEC JG280 or in *E. coli* DH5α was also examined in the absence or presence of isolate LB1. Clones DH5α-STa and DH5α-LT and JG280 expressed a similar level of *estA* and *elt in vitro* (Fig. 6A). However, the expression of *estB* was increased to more than 2-fold in *E. coli* DH5α than in ETEC JG280 in the absence of LB1. In the presence of LB1, approximate 40% and 60% reduction in the expression of all enterotoxin genes (*estA*, *estB*, and *elt*) were observed in *E. coli* DH5α and in ETEC JG280, respectively, compared with the absence of LB1 (Fig. 6B).

## Discussion

A number of pathogens of human and animal origin have been studied using *C. elegans* to model the pathogen and host interaction [4]–[10]. The present study is the first report, to our best knowledge, describing the establishment of the *C. elegans* assay model to measure the response of the nematode to ETEC infection. The establishment of the life-span assay model has enabled not only an effective preselection of probiotic candidates that have potential to control ETEC infection, but more so the studies on the mechanism underlying the protection provided by the candidate isolates. The results have identified several promising isolates highly protective to the nematode for further *in-vivo* studies on pigs to be challenged with ETEC. In addition, we have determined that the protection offered by *Lactobacillus* to *C. elegans* was not from inhibition of intestinal colonization of ETEC, but from suppression of its *in-vivo* gene expression of enterotoxins, particularly heat-stable toxins (STa and STb), which were mainly responsible for the nematode death after ETEC infection.

The intestinal colonization of ETEC is usually associated with animal diarrhea [27]–[29]. Binding of fimbrial adhesins to animal intestines is an essential step of ETEC pathogenesis that causes diarrhea in the host animal. It is also clear, based on previous studies, that ETEC growth and attachment in animal intestines can be reduced by *Lactobacillus* [30]–[32]. In the present study, we initially attempted to determine if inhibition of ETEC intestinal colonization by *Lactobacillus* was responsible for the protection. The results from bacterial enumeration by plating indicated that the level of ETEC strain JG280 in the *C. elegans* intestine during the life-span assays was approximately 5×10^4 ^CFU/worm and the level was not significantly affected by *Lactobacillus* treatment, with either strong or weak protectors. Further analysis with TEM also supported this notion, which demonstrated that ETEC JG180 and *L. zeae* LB1 cells were able to co-exist in the intestine of *C. elegans* (Fig. 3). These results clearly suggest that inhibition of ETEC intestinal colonization was not the mechanism through which *Lactobacillus* provided protection to *C. elegans*.

ETEC-induced diarrhea is caused mainly by enterotoxins ST and LT produced by ETEC, which can affect Cl^-^ and Na^+^ secretion and cGMP levels in host cells [33]–[35]. Previous *in-vitro* studies have shown that some *Lactobacillus* isolates were able to protect animal tissue cultures by inhibiting the production of ETEC enterotoxins [36], [37]. In the present study, K88^+^ strain JFF4, which is lack of enterotoxin genes (*estA*, *estB*, and *elt*), was unable to kill *C. elegans* in the life-span assay (Fig. 1). The gene expression of the three enterotoxins was significantly increased during ETEC JG280 infection of *C. elegans*, which was coincident with the death of the worms (Fig. 4). The gene expression was, however, remarkably reduced by the treatment with *L. zeae* LB1 that was able to effectively protect the nematode from death. On the other hand, the two heat-stable enterotoxin clones (DH5α-STa and DH5α-STb) were as effective as ETEC JG280 in killing *C. elegans*, although the heat-labile toxin clone (DH5α-LT) killed only approximately 40% of the worms (Fig. 5A). The treatment with *L. zeae* LB1 also prevented the nematode from death caused by individual enterotoxin clones (Fig. 5B). Moreover, gene expression of the enterotoxins in either ETECT JG280 or *E. coli* DH5α was partially suppressed by treatment with *L. zeae* LB1 *in vitro* (Fig. 6B), which appears to support the role of *Lactobacillus in-vivo* in controlling enterotoxin gene expression. In view of these results, it appears that the enterotoxins from ETEC JG280 were mainly responsible for the death of *C. elegans* caused by infection and the protection of the nematode by *Lactobacillus* was achieved mainly through inhibition of the enterotoxin gene expression.

It has been well documented that *Lactobacillus* can induce the host immune response [38]. *L. rhamnosus* LGG has been reported to prevent ETEC JG280-induced diarrhea by enhancing immune response of pigs [39]. *Lactobacillus* can also protect host animals by regulating chemokine and cytokine gene expression [40]. Moreover, improvement in the maintenance of membrane barrier integrity can be another potential mechanism [41]. Although *C. elegans* is lack of an adaptive immune system, it possesses three major signaling pathways in its defense system, including its anti-microbial response [42]. These pathways include: 1) P38-MAPK pathway, 2) TGF-β pathway, and 3) Insulin/IGF-like pathway. *Lactobacillus* can also induce *C. elegans*’s host response [43], [44]. In the present study, *L. zeae* LB1 was able to effectively prevent *C. elegans* from death caused by ETEC JG280 or individual enterotoxin clones (Table 1, Fig. 2 and Fig. 5). While the gene expression of enterotoxins was significantly reduced (several folds or more) by *L. zeae* LB1 *in vivo* (Fig. 4), the inhibition by the same isolate was about 40% *in vitro* (Fig. 6B), implying the involvement of host factors that may have contributed to the protection induced by *Lactobacillus*. To clarify the issue, further studies are required.

In conclusion, the present study has established a *C. elegans* life-span assay that has enabled a measurement of the nematode response to ETEC infection. Using this assay model, several LAB isolates with probiotic potential to develop into probiotics have been identified for further ETEC challenge studies on pigs. Of greater interest, inhibition of enterotoxin gene expression, rather than interference of ETEC in intestinal colonization, during the ETEC infection, was identified as the potential mechanism through which *Lactobacillus* protected *C. elegans*.

## Materials and Methods

### *C. elegans* and Bacteria

In the present study, the SS104 strain of *C. elegans* harboring a temperature-sensitive allele of *glp-4 (bn2)* was used (Genetics Center, University of Minnesota, Minnepolis). The strain is able to produce progeny at 15°C but not at 25°C; therefore, it could be maintained at 15°C and cultivate at 25°C for life-span assays. The standard procedures for maintenance and synchronization of all general worms were previously described [17]. *E. coli* OP50 grown in Luria-Bertani broth or agar at 37°C for 12 h with a cell density of 10^8^ Colony Forming Unit (CFU) per milliliter was used as food for the nematode. Nematode growth medium (NGM), S medium, and M9 buffer were used for culturing and *C. elegans* life-span assays [17].

K88^+^ ETEC strain JG280 is a haemolytic *E. coli* of serotype O149: K88 (F4), a porcine isolate possessing the toxin genes of *elt*, *estA*, *estB*, and *astA*, and antibiotic resistance to tetracycline, ceftiofur, ampicillin, spectinomycin, apramycin, gentamicin, neomycin, and trimethoprim/sulfonamide [18]. K88^+^ strain JFF4 is also a porcine isolate having F4/K88 fimbriae but lacking enterotoxin genes of *estA*, *estB,* and *elt* [19]. *Lactobacillus* isolates were originally obtained from the adult pig or chicken intestine and some of them have been reported previously [7]. Collection of intestinal digesta and fecal samples from chickens and pigs was conducted following the protocols (#05R053, #09R104) approved by the Animal Care and Use Committee of the University of Guelph for the use of animals. Either de Man Rogosa Sharpe (MRS) broth or agar was used to culture *Lactobacillus* isolates at 37°C for 18–24 h in an anaerobic chamber (Coy Laboratory Products, Grass Lake, MI) with an atmosphere of 85% N_2_, 10% CO_2_, and 5% H_2_.

### Life-span Assay of *C. elegans*

The duration of the *C. elegans* assay with various bacterial treatments was performed as described previously [7]. In brief, to synchronize *C. elegans* to the same age and stage, gravid adult worms were treated with sterile water containing 0.5 M NaOH and a 0.5% freshly prepared NaClO solution. The eggs were released and isolated by centrifugation (1,300×*g* for 1 min) and then re-suspended in M9 buffer, and then hatched at 20°C for 16 h. The L1 larvae were transferred to NGM agar with *E. coli* OP50 at 25°C for 48–60 h until they reached the L4 stage. After collecting the worms from the NGM plate with M9 buffer and washing three times in the S medium via centrifugation (1,300×*g* for 2 min) and suspension, 15–20 worms were transferred to each well of a 24-well plate containing 2 ml of S medium and then incubated at 25°C. The assays generally lasted for 7 to 10 days.

To establish the killing assay with ETEC strain JG280, different concentrations of JG280 (10^7^–10^9^ CFU/ml) were incubated with worms for 10 days. Worms fed only *E. coli* OP50 served as the negative control. For the killing assays with individual enterotoxin clones, each clone was tested at the same concentration (2×10^8^ CFU/ml) as measured in the assays with JG280. In the assays to evaluate the effect of a LAB isolate on protecting the nematode from death caused by JG280 or by the enterotoxin clones, the first day that worms were fed *E. coli* OP50 (10^8^ CFU/ml) or individual LAB isolates (10^9^ CFU/ml) was designated as day 0. After 18 h incubation, the worms were collected and washed three times in the S medium via centrifugation and suspension. The assays were started by mixing the washed worms with JG280 or individual enterotoxin clones at the final concentration of 2×10^8^ CFU/ml in a 24-well plate, which was designated as day 1. The inclusion of *E. coli* OP50 (10^5 ^CFU/ml) in the assays (from day 1 to the end of assays) was also tested and no significant effect on the survival of nematode, compared to the assays without supplement of *E. coli* OP50, was observed. Worms treated with *E. coli* OP50 only served as the negative control and worms treated with *E. coli* OP50 (18 h) and then with JG280 were regarded as the ETEC reference group. The volume of the assay mixture in each well was 2 ml. All of the bacterial cultures used for the *C. elegans* life-span assays, regardless of their origins, were in the early stationary phase. They were all washed twice in the S medium by centrifugation and suspension before addition to 24-well plates. To determine the survival rate of *C. elegans*, the number of live worms was recorded daily, and the percentage of surviving worms was calculated by the following formula: survival (%) = (live worms/total worms used) ×100. A worm was considered to be dead when it failed to respond to touch.

### Examination of Bacterial Colonization in the Intestine of *C. elegans*

The numbers of JG280 cells in the nematodes intestine were determined with little modification of the method described previously [7]. Worms were incubated with *E. coli* OP50, JG280, or a *Lactobacillus* isolate followed by sampling (10 worms per sample) every 2 days until the end of assays. Sampled worms were washed twice in the M9 buffer, treated with 0.6 ml of 70% ethanol for 20 s for surface sterilization, and then mixed with 6 ml of M9 buffer immediately with the final concentration of ethanol reduced to approximately 6.4%. After two more washes with the M9 buffer, these surface-sterilized worms were examined. They had intact bodies, and no growth of bacteria was apparent on the nutrient agar plates, indicating that no live bacterial cells were associated with the surface of the worms (data not shown). After surface sterilization, the worms were mashed mechanically with a pellet pestle motor, re-suspended in the M9 buffer, and plated on LB with antibiotics (15 µg/ml erythromycin, 30 µg/ml chloramphenicol, 60 µg/ml streptomycin), or MRS agar for counting of *E. coli* OP50, ETEC JG280 and *Lactobacillus*, respectively.

### RNA Extraction

#### *In-vitro* experiment

In the experiment to examine *in-vitro* expression of enterotoxins in ETEC JG280 and in *E. coli* DH5α, the bacteria were grown in the LB medium at 37°C for 18 h followed by 4 h incubation in the absence or presence of *L. zeae* LB1 in the S medium at 25°C. Bacterial cells were then harvested and the total RNA was extracted by using the mirVana miRNA Isolation Kit (Ambion, TX) according to manufacturer’s instructions.

#### *In-vivo* experiment

Total RNA of bacteria and *C. elegans* from the life span assays was extracted from lysates of the worms, which harbored bacterial cells in their intestine due to the treatments with JG280 or with both *Lactobacillus* and JG280, using the mirVana miRNA Isolation Kit (Ambion, TX) according to manufacturer’s instructions. To prepare the lysates, about five thousand worms that had been stored in RNA*later* Solution (Ambion, TX) following by 2 washes with PBS immediately after sampling were disrupted in 0.8 ml of a Lysis/Binding Buffer (mirVana miRNA Isolation Kit) by a bead-beater (PowerLzyer24, MO BIO Laboratories, Inc., Carlsbad, CA). The beating was conducted at 3,500 rpm for two cycles followed by four cycles at 3,000 rpm and four cycles at 2,500 rpm. Each cycle lasted for 1.5 min and there was a 2 min interval between two cycles with the samples on ice. After RNA extraction, the samples were treated with DNase I (Ambion, TX) at 37°C for 30 min and then verified as DNA-free by PCR assays with primers specific to glyceraldehyde-3-phosphate dehydrogenase (*gapA*). RNA integrity was determined by visualization in an agarose gel [20]. The concentration of total RNA was determined with a NanoDrop ND-1000 spectrophotometer (NanoDrop Technologies, Wilmington, DE).

### Reverse Transcription and Real-time QPCR Analysis

Bacterial gene expression was determined by reverse transcription and quantitative PCR (QPCR) analysis as described previously [21] with some modifications. Briefly, a RNA sample was used for first-strand cDNA synthesis using the SuperScript first-strand synthesis system (Invitrogen, Carlsbad, CA) according to manufacturer’s instructions. Housekeeping gene *gapA* was used to normalize input amounts of RNA and the levels of *estA*, *estB*, and *elt* expressed. QPCR was subsequently performed using a Stratagene MX3005 thermal cycler and brilliant SYBR green QPCR master mix (Bio-Rad Laboratories, Richmond, VA). Previously published PCR primers specific to each of *estA* (STa), *estB* (STb), *elt* (LT), and *gapA* genes (Table S2) [23] were experimentally validated and used for QPCR assays. One µl of each cDNA sample was included in a 24 µl reaction mixture containing 12.5 µl Master Mix, 3.75 µl each of the primers at 150 nM, and 4 µl irradiated and double autoclaved dH_2_O. The QPCR programs included 10 min at 95°C and 40 cycles of 95°C for 30 s, 56°C for 1 min, and 72°C for 30 s. Fluorescence was measured after each annealing during the cycles.

QPCR data were analyzed using the 2^−ΔΔCt^ method to determine the relative abundance (fold changes) of target genes [22]. The cycle threshold, Ct, is the point at which fluorescence above the background is statistically significant. Ct values were determined with the MX3005 software based on a threshold line that was manually defined above the non-informative fluorescent data. ΔCt represents the difference between the Ct value with the primers to a target gene and the Ct value to the housekeeping gene (*gapA*). ΔΔCt represents the difference between the ΔCt value of each time point after incubation and the ΔCt value of zero time point. The values derived from 2^−ΔΔCt^ represent fold changes of samples in abundance relative to the reference samples. The reference samples (zero time point) had the 2^−ΔΔCt^ value of 1. In the experiment to examine *in- vitro* gene expression of enterotoxins, 2^−ΔCt^ was used to represent relative expression of enterotoxins to the housekeeping gene (Fig. 6).

### Transmission Electron Microscopy

*C. elegans* infected with ETEC JG280 following *L. zeae* LB1 or no treatment was collected on day 2 post infection and used for thin-section preparation and electron microscopy analysis. First, the worms were fixed in 2.5% glutaraldehyde at 22°C for 16 h, rinsed several times in 0.1 M cacodylate buffer, and then re-stained in 1.0% (wt/vol) osmium tetroxide in 0.1 M cacodylate buffer for 1 h at 22°C. After the samples were transferred to double distilled water, they were stained, en bloc in 2.0% aqueous uranyl acetate for 1 h at 22°C and then embedded in Epon and LR Gold as described [26]. The images were viewed using a Philips CM10 equipped with a top mount SIS/Olympus Morada 11 megapixel CCD camera.

### Cloning of Enterotoxin Genes from ETEC JG280

PCR primers used for cloning enterotoxin genes were designed based on the *E. coli* MG1655 genome sequences in the NCBI database and are listed in Table S2 [24], [25]. The high-fidelity PCR using F-530L Phusion DNA Polymerase (BioLab, New England) was conducted with genomic DNA of strain JG280 as the template. The PCR program consisted of a 4-min initiation at 94°C for denaturing the template DNA and 32 cycles of 94°C for 30 s, 56°C for 30 s, and 72°C for 1–2 min for amplification (depending on the size of the target gene) followed by a final extension at 72°C for 10 min and another 20 min at 72°C with Taq DNA polymerase to add an A-tail. The PCR amplicons were cloned into the pCR4-TOPO vector according to the manufacturer’s instructions (TOPO TA cloning kit; Invitrogen, Carlsbad, CA). The colonies with recombinant pCR4 plasmids were selected by plating the transformants on Luria broth agar plates containing 100 µg/ml ampcilin. Positive clones containing a recombinant pCR4 plasmid with the target gene were determined by PCR using a pair of cloning primers listed in Table S2 [23]–[25]. Sequence and its orientation of the clones confirmed by PCR were examined by sequencing. Cloned *estA*, *estB*, and *elt* genes were identical in their sequences (100% homology) to the three toxin genes of ETEC strain JG280, based on the NCBI blast analysis.

### Statistical Analysis

All statistical computation analyses were performed using the Statistical Analysis System (SAS release 9.2, SAS Institute Inc., Cary, NC). Survival curves for *C. elegans* were compared using the Kaplan-Meier survival analysis followed by a log-rank test. One-way analysis of variance (ANOVA) and the Tukey’s multiple comparisons were carried out to test for significant differences between the means. Means with *P* values of ≤0.05 were considered to differ significantly.

## Supporting Information

Table S1 ***E. coli*** **strains.**
(DOCX)

Table S2 **Cloning and QPCR primers.**
(DOCX)
