# Supplementary material for: Lactobacillus zeae Protects Caenorhabditis elegans from Enterotoxigenic Escherichia coli-Caused Death by Inhibiting Enterotoxin Gene Expression of the Pathogen
Source: PLoS One. 2014 Feb 18;9(2):e89004. doi: 10.1371/journal.pone.0089004 (PMC3928337; doi:10.1371/journal.pone.0089004)
Supplement: Table S1 — E. coli strains. (DOCX) [file pone.0089004.s001.docx]

**Table S1. *E. coli* strains**

| Strain | Description | Source or Reference |
| --- | --- | --- |
|  |  |  |
| JG280 | ETEC O149: K88(F4) strain, positive for K88, *elt*, *estA*, and *estB* | 18 |
| JFF4 | *E. coli* strain, positive for K88 but negative for *elt*, *estA*, and *estB* | 19 |
| DH5α-T1^R^ | *E. coli* DH5α-T1^R^ harboring plasmid pCR^®^4-TOPO^®^, used as a cloning host | Invitrogen |
| DH5α-STa | *E. coli* DH5α-T1^R^ harboring enterotoxin gene *estA* in plasmid pCR^®^4-TOPO^®^ | This study |
| DH5α-STb | *E. coli* DH5α-T1^R^ harboring enterotoxin gene *estB* in plasmid pCR^®^4-TOPO^®^ | This study |
| DH5α-LT | *E. coli* DH5α-T1^R^ harboring enterotoxin gene *elt* in plasmid pCR^®^4-TOPO^®^ | This study |
| DH5α-opSTa | *E. coli* DH5α-T1^R^ harboring enterotoxin gene *estA* with a opposite orientation in plasmid pCR^®^4-TOPO^®^ | This study |
| DH5α-opLT | *E. coli* DH5α-T1^R^ harboring enterotoxin gene *elt* with a opppsite orientation in plasmid pCR^®^4-TOPO^®^ | This study |
| DH5α-16SrRNA | *E. coli* DH5α-T1^R^ harboring a part of 16SrRNA gene from *E. coli* in plasmid pCR^®^4-TOPO^®^ | This study |
