# Supplementary material for: Lactobacillus zeae Protects Caenorhabditis elegans from Enterotoxigenic Escherichia coli-Caused Death by Inhibiting Enterotoxin Gene Expression of the Pathogen
Source: PLoS One. 2014 Feb 18;9(2):e89004. doi: 10.1371/journal.pone.0089004 (PMC3928337; doi:10.1371/journal.pone.0089004)
Supplement: Table S2 — Cloning and QPCR primers. (DOCX) [file pone.0089004.s002.docx]

**Table S2. Cloning and QPCR primers**

| Primer | Amplicon (bp) | Sequence (5' to 3') | Source or Reference |
| --- | --- | --- | --- |
| **For QPCR** |  |  |  |
| *estA*-F | 158 | CAACTGAATCACTTGACTCTT | 23 |
| *estA*-R |  | TTAATAACATCCAGCACAGG |  |
| *estB*-F | 113 | TGCCTATGCATCTACACAAT | 23 |
| *estB*-R |  | CTCCAGCAGTACCATCTCTA |  |
| *elt*-F | 322 | TCTCTATGTGCATACGGAGC | 23 |
| *elt*-R |  | CCATACTGATTGCCGCAAT |  |
| *gapA*-F | 299 | TCCGTGCTGCTCAGAAACG | 23 |
| *gapA*-R |  | CACTTTCTTCGCACCAGCG |  |
| **For cloning** |  |  |  |
| *estA*-14590 F | 589 | TAGTCATCCTACCATCCTGC | This study |
| *estA*-15178 R |  | GCCTCATCTGAACCGTCTGG |  |
| *estB*-290 F | 496 | ACGGGTGATTGACACTACAC | This study |
| *estB*-785 R |  | ACATTGTCCATTTCTCACTGCA |  |
| *elt*-16612 F | 1275 | CGGATTGTCTTCTTGTATGAT | 24 |
| *elt*-17886 R |  | GATCGGTATTGCCTCCTCTAC |  |
| E16s RNA-8 F | 527 | AGAGTTTGATCCTGGCTCAG | 25 |
| E16s RNA-534 R |  | ATTACCGCGGCTGCTGGC |  |

The cloning primer pair for *estA* was designed based on the sequence of gene with GenBank Accession# CP000913 and the pair for *estB* was based on the sequence of gene with GenBank Accession# M35586.
